# Instruments Used in the Extraction of Teeth

**Published:** 1876-01

**Authors:** S. P. Cutler

**Affiliations:** Memphis, Tenn.


### INSTRUMENTS USED IN THE EXTRACTION OF TEETH.

BY S. P. CUTLER, M D., D. D. S., MEMPHIS, TENN.

If I should attempt to criticise the instruments in common use, and in the various dental depots, made by the best makers on the most approved pattern, it would comprise an extended essay alone. Of all I have seen in the various depots, there may be one in ten or twenty properly constructed to fill all the requirements pertaining thereto.

As to what constitutes a complete set of extractors, there is diversity of opinion. Some dentists having not more than five or six forceps and two or three elevators, for all cases; while others require forty or fifty, or more, instruments for a full set; myself among the latter.

For the upper adult molars I have eight forceps, right and left, of different sizes, similar pattern, one set of "cow horns." Two wisdom forceps, upper of different patterns, one bicuspid forcep, superior, for right and left, these forceps answer equally for cuspid, central incisors and in some cases lateral. I have five or six different forceps for the front teeth, of various patterns and sizes.

For the lower molars, I have right and left, three forceps for either side, six in all, one "cow horn" for either side, three lower wisdom forceps, for either side, besides the elevator forceps; one bicuspid which may be used for cuspid and sometimes incisors, several sizes and patterns for the lower front teeth proper.

Besides these, to complete, I have a number of fang forceps, of various shapes and sizes, for above and below. These constitute my set of forceps, which are all good, some of them of my own design, most of them have been altered to suit my ideas. They are of various makes, some thirty or forty years old, some quite recent. All the old ones were very faulty generally; the points were all too long, one great error, the fulcrumage being out of proportion to leverage, the spring or elasticity in the wrong place. Many defects pertaining to the older make have been remedied to a great extent, though not yet perfect. In constructing forceps, the handles, joints and points, should be in relative proportion, so that there will be a certain amount of spring or elasticity, without any giving away or slipping on the tooth, and still not too unyielding, so as not to give it a dead lock or grip, which fails to indicate the exact amount of force being exerted, and danger of crushing, and prevents the operator from judging to what extent the tooth is being loosened or giving away in the socket.

The great point in extracting teeth of all classes, is to know precisely what the instrument is doing.

In most of our molar forceps, the points come too close together, and the handles at their ends, too wide apart, the opposite should be the case, so that an easy grasp and firm grip may be had. Some hands are short, others long, and the same forceps will not suit both extremes equally well. The middle average, as to the handles, comes nearer being satisfactory in this respect. There is a great variety of other appliances to complete a set, such as elevators, grubbing hoes, hooks, pushers, pullers, etc., etc. Screws, of different sizes and tapes, all come in to complete a set.

The points in all of these should be made as thin and delicate as possible, without sacrificing strength. Almost all instruments found in the depots are faulty in this respect.

Whenever fangs are to be extracted, whenever elevators are admissible, they are preferable, give less pain and are more reliable in general, when properly used.

With a full and complete set of instruments at hand, we often meet with cases that puzzle our best skill, from the fact that we lack the precise instrument. There is generally too much force or strength used, thereby increasing the danger of breaking something.

Breaking teeth, and failure to succeed now a days, is wholly out of the question, such a thing is unprofessional, except in extreme cases, or where the patient is the cause..

When a tooth, or teeth are to be extracted, the best plan is to first diagnose the case well before attempting to extract, then select the proper instrument and place it in the most convenient place, without any flourish or parade at all. Next have the patient in the best possible position, before taking the instrument in hand, then proceed with proper dispatch in a business like way, and place the instrument on the tooth without being nervous or excited at all, then push the instrument home, or as far upon the tooth as possible before making any attempt to extract.

Your own body and all your muscles should also be in the best possible relation to your patient. I prefer, as a rule, to stand on the right side, a little behind my patient, his head thrown back, the left arm around the head and the fingers of the same manipulating the mouth to suit the case, mouth wide open.

You may now consider yourself master of the situation, confident and self-reliant, assuring your patient of success and inspire confidence. No parade or flourish of trumpets, no weak nerves. Right here more address and firmness is necessary than in any other dental operation.

No gum lancet is necessary only in exceptional cases. Now the knight of the forceps must be the hero of the bloody carnage, as it is all on one side, and no enemy in sight, (*Cui bono.*)

Your grip being firm, you gently rotate the forceps on all single fang teeth, even molars, in many instances, the tooth comes away without any pulling. Some bicuspids will not thus easily come away and must be shaken by swaying the hand in and out, until the connection is broken up, then pull if necessary with a rocking motion; never a straight pull under any circumstances. During all this time the forceps should be pushed up the fang, until the connection is severed, by so pressing till the points pass some distance up into the socket and wedges out the alveolus from the tooth, and in case of molars the points pressing the alveolus, acts as a fulcrum, increasing the leverage and is a safety against accidents.

As a rule, much more muscular force is exerted in extractions than is necessary, there is a slight of hand acquired by practice and study, unknown to the tyro.

Permit me to state, that there is as much bad tooth pulling as anything else in dentistry.

In using elevators, I often press up the blade with the fingers of the left hand, and keep them on the same while removing the fang to assist, also to avoid accidents.

